# Bis[μ-*N*′-(5-bromo-3-meth­oxy-2-oxido­benzyl­idene)-2-hydroxybenzohydra­zidato]bis[(*N*,*N*-dimethyl­formamide)­copper(II)]

**DOI:** 10.1107/S1600536812036100

**Published:** 2012-08-25

**Authors:** Shunsheng Zhao, Lanlan Li, Xiangrong Liu, Weixu Feng, Xingqiang Lü

**Affiliations:** a College of Chemistry and Chemical Engineering, Xi’an University of Science and Technology, Xi’an 710054, Shaanxi, People’s Republic of China; b College of Chemical Engineering, Northwest University, Xi’an 710069, Shaanxi, People’s Republic of China

## Abstract

The title compound, [Cu_2_(C_15_H_11_BrN_2_O_4_)_2_(C_3_H_7_NO)_2_], is derived from the reaction of *N*′-(5-bromo-2-hy­droxy-3-meth­oxy­benzyl­idene)-2-hy­droxy­benzohydrazide and copper nitrate in a dimethyl­formamide solution in the presence of sodium hydroxide. The compound can be regarded as a binuclear centrosymmetric complex. In the crystal, the Cu^II^ atom is fivefold surrounded and adopts a distorted square-pyramidal coordination environment. An intra­molecular O—H⋯N hydrogen bond stabilizes the mol­ecular conformation.

## Related literature

For the synthesis of *N*′-(5-bromo-2-hy­droxy-3-meth­oxy­benzyl­idene)-2-hy­droxy­benzohydrazide and its crystal structure, see: Zhao *et al.* (2012 ▶). For the crystal structure of a complex with a similar coordination environment, see: Huang *et al.* (2010 ▶).
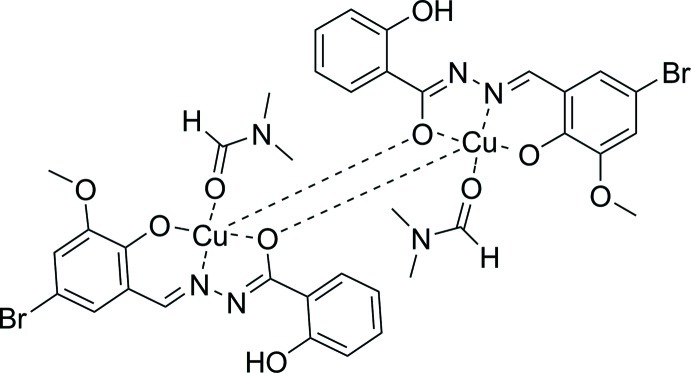


## Experimental

### 

#### Crystal data

[Cu_2_(C_15_H_11_BrN_2_O_4_)_2_(C_3_H_7_NO)_2_]
*M* *_r_* = 999.61
Triclinic, 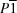

*a* = 8.3861 (17) Å
*b* = 9.5795 (19) Å
*c* = 12.275 (3) Å
α = 90.446 (3)°
β = 97.850 (3)°
γ = 101.688 (3)°
*V* = 956.0 (3) Å^3^
*Z* = 1
Mo *K*α radiation
μ = 3.27 mm^−1^
*T* = 296 K
0.38 × 0.25 × 0.16 mm

#### Data collection

Bruker SMART 1K CCD area-detector diffractometer
Absorption correction: multi-scan (*SADABS*; Sheldrick, 2004 ▶) *T*_min_ = 0.371, *T*_max_ = 0.620
5880 measured reflections
4354 independent reflections
2957 reflections with *I* > 2σ(*I*)
*R*_int_ = 0.022

#### Refinement

*R*[*F* ^2^ > 2σ(*F* ^2^)] = 0.040
*wR*(*F* ^2^) = 0.123
*S* = 0.99
4354 reflections
256 parameters
H-atom parameters constrained
Δρ_max_ = 0.59 e Å^−3^
Δρ_min_ = −0.58 e Å^−3^

### 

Data collection: *SMART* (Bruker, 2001 ▶); cell refinement: *SAINT* (Bruker, 2001 ▶); data reduction: *SAINT*; program(s) used to solve structure: *SHELXS97* (Sheldrick, 2008 ▶); program(s) used to refine structure: *SHELXL97* (Sheldrick, 2008 ▶); molecular graphics: *SHELXTL* (Sheldrick, 2008 ▶); software used to prepare material for publication: *SHELXTL* and local programs.

## Supplementary Material

Crystal structure: contains datablock(s) I, global. DOI: 10.1107/S1600536812036100/bt5995sup1.cif

Structure factors: contains datablock(s) I. DOI: 10.1107/S1600536812036100/bt5995Isup2.hkl

Additional supplementary materials:  crystallographic information; 3D view; checkCIF report

## Figures and Tables

**Table 1:** Hydrogen-bond geometry (Å, °)

*D*—H⋯*A*	*D*—H	H⋯*A*	*D*⋯*A*	*D*—H⋯*A*
O4—H4*A*⋯N2	0.82	1.84	2.566 (3)	146

**Fig. 1.**
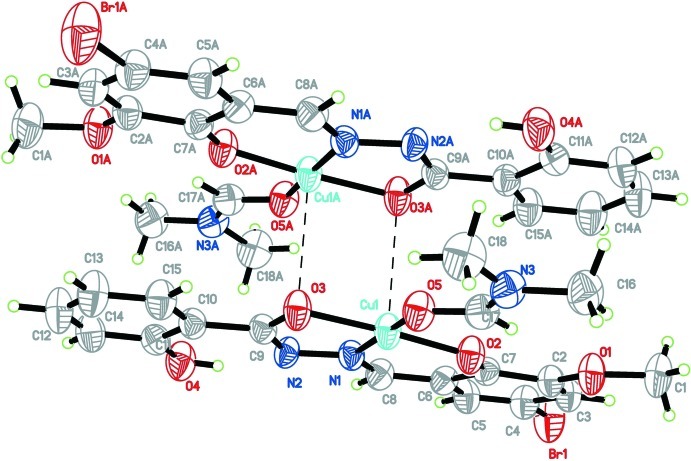
The molecular structure of the title compound, with atom labels and 50% probability displacement ellipsoids for non-H atoms.

## supplementary crystallographic information

### Comment

Hydrazones attract the interest of researchers due to their various biological activities and their capacity for chelating to most kind of metals. As Fig. 1 shows, the Cu_II_ ion exists in a distorted square-pyramidal coordination geometry and it is located in the center of the coordination basal plane, which is defined by three donor atoms (O2, N1 and O3) of the hydrozone ligand and O5 atom from the DMF molecule with a mean plane deviation of 0.0367 (4) Å. The axial position is occupied by O3 atom from another asymmetric unit. The molecular conformation is stabilized by an intramolecular O—H···N hydrogen bond (Table 1).

### Experimental

A solution of copper nitrate (186.2 mg, 1.0 mmol) in DMF (2 ml) was added to a solution of *N*'-(5-bromo-2-hydroxy-3-methoxybenzylidene)-2-hydroxybenzohydrazide (361.5 mg, 1.0 mmol) in DMF (10 ml) and stirred at room temperature for 2 h before being filtered. The dark green filtrate was allow to evaperate slowly in the air for several days. Green crystals was collected by filtration and dried under vacumn, yield 59.3%.

### Refinement

H atoms were positioned geometrically and refined using a riding model with C—H = 0.95–0.99 Å, O—H = 0.82 Å and with *U*_iso_(H) = 1.2 *U*_eq_(C,O) (1.5 for methyl groups and the hydroxyl group). The methyl groups bonded to N and the hydroxyl group were allowed to rotate but not to tip.

### Crystal data

**Table:**

[Cu_2_(C_15_H_11_BrN_2_O_4_)_2_(C_3_H_7_NO)_2_]	*Z* = 1
*M**_r_* = 999.61	*F*(000) = 502
Triclinic, *P*1	*D*_x_ = 1.736 Mg m^−^^3^
Hall symbol: -P 1	Mo *K*α radiation, λ = 0.71073 Å
*a* = 8.3861 (17) Å	Cell parameters from 3650 reflections
*b* = 9.5795 (19) Å	θ = 1.8–26.5°
*c* = 12.275 (3) Å	µ = 3.27 mm^−^^1^
α = 90.446 (3)°	*T* = 296 K
β = 97.850 (3)°	Block, green
γ = 101.688 (3)°	0.38 × 0.25 × 0.16 mm
*V* = 956.0 (3) Å^3^	

### Data collection

**Table:**

Bruker SMART 1K CCD area-detector diffractometer	4354 independent reflections
Radiation source: fine-focus sealed tube	2957 reflections with *I* > 2σ(*I*)
Graphite monochromator	*R*_int_ = 0.022
thin–slice ω scans	θ_max_ = 29.5°, θ_min_ = 2.7°
Absorption correction: multi-scan (*SADABS*; Sheldrick, 2004)	*h* = −10→9
*T*_min_ = 0.371, *T*_max_ = 0.620	*k* = −13→9
5880 measured reflections	*l* = −15→16

### Refinement

**Table:**

Refinement on *F*^2^	Primary atom site location: structure-invariant direct methods
Least-squares matrix: full	Secondary atom site location: difference Fourier map
*R*[*F*^2^ > 2σ(*F*^2^)] = 0.040	Hydrogen site location: inferred from neighbouring sites
*wR*(*F*^2^) = 0.123	H-atom parameters constrained
*S* = 0.99	*w* = 1/[σ^2^(*F*_o_^2^) + (0.069*P*)^2^] where *P* = (*F*_o_^2^ + 2*F*_c_^2^)/3
4354 reflections	(Δ/σ)_max_ = 0.001
256 parameters	Δρ_max_ = 0.59 e Å^−^^3^
0 restraints	Δρ_min_ = −0.58 e Å^−^^3^

### Special details

**Table:**

Geometry. All e.s.d.'s (except the e.s.d. in the dihedral angle between two l.s. planes) are estimated using the full covariance matrix. The cell e.s.d.'s are taken into account individually in the estimation of e.s.d.'s in distances, angles and torsion angles; correlations between e.s.d.'s in cell parameters are only used when they are defined by crystal symmetry. An approximate (isotropic) treatment of cell e.s.d.'s is used for estimating e.s.d.'s involving l.s. planes.
Refinement. Refinement of *F*^2^ against ALL reflections. The weighted *R*-factor *wR* and goodness of fit *S* are based on *F*^2^, conventional *R*-factors *R* are based on *F*, with *F* set to zero for negative *F*^2^. The threshold expression of *F*^2^ > σ(*F*^2^) is used only for calculating *R*-factors(gt) *etc*. and is not relevant to the choice of reflections for refinement. *R*-factors based on *F*^2^ are statistically about twice as large as those based on *F*, and *R*- factors based on ALL data will be even larger.

### Fractional atomic coordinates and isotropic or equivalent isotropic displacement parameters (Å^2^)

**Table:**

	*x*	*y*	*z*	*U*_iso_*/*U*_eq_	
Cu1	0.08579 (5)	0.69096 (4)	1.00600 (3)	0.04176 (15)	
Br1	−0.38768 (6)	1.16095 (5)	1.23139 (4)	0.0821 (2)	
O2	0.1023 (3)	0.8271 (2)	1.11968 (18)	0.0456 (6)	
O3	0.0529 (3)	0.5575 (2)	0.88176 (17)	0.0472 (6)	
N1	−0.1145 (3)	0.7314 (3)	0.9327 (2)	0.0383 (6)	
C8	−0.1977 (4)	0.8182 (4)	0.9650 (3)	0.0435 (8)	
H8A	−0.2920	0.8293	0.9194	0.052*	
N2	−0.1691 (3)	0.6616 (3)	0.8310 (2)	0.0397 (6)	
O4	−0.3540 (3)	0.5853 (3)	0.6472 (2)	0.0592 (7)	
H4A	−0.3231	0.6295	0.7064	0.089*	
O1	0.1737 (3)	0.9882 (3)	1.29675 (19)	0.0532 (6)	
C7	−0.0064 (4)	0.8995 (3)	1.1382 (2)	0.0373 (7)	
C5	−0.2652 (4)	0.9817 (4)	1.0956 (3)	0.0473 (8)	
H5A	−0.3618	0.9825	1.0485	0.057*	
C2	0.0244 (4)	0.9885 (4)	1.2357 (3)	0.0427 (8)	
C15	−0.0090 (4)	0.4028 (4)	0.6826 (3)	0.0503 (9)	
H15A	0.0814	0.3948	0.7334	0.060*	
C9	−0.0709 (4)	0.5740 (3)	0.8131 (2)	0.0378 (7)	
C10	−0.1099 (4)	0.4926 (3)	0.7074 (3)	0.0404 (7)	
C3	−0.0851 (4)	1.0652 (4)	1.2625 (3)	0.0463 (8)	
H3A	−0.0634	1.1207	1.3274	0.056*	
C13	−0.1735 (5)	0.3340 (5)	0.5115 (3)	0.0665 (11)	
H13A	−0.1945	0.2805	0.4456	0.080*	
C11	−0.2471 (4)	0.5005 (4)	0.6306 (3)	0.0442 (8)	
C4	−0.2312 (5)	1.0593 (4)	1.1908 (3)	0.0501 (9)	
C12	−0.2778 (5)	0.4198 (4)	0.5330 (3)	0.0595 (10)	
H12A	−0.3695	0.4240	0.4822	0.071*	
C6	−0.1546 (4)	0.8990 (3)	1.0669 (3)	0.0396 (7)	
C14	−0.0388 (5)	0.3255 (5)	0.5852 (3)	0.0641 (11)	
H14A	0.0322	0.2676	0.5694	0.077*	
O5	0.2998 (3)	0.6509 (2)	1.06249 (19)	0.0480 (6)	
N3	0.5109 (3)	0.6665 (3)	1.1985 (2)	0.0469 (7)	
C17	0.3796 (4)	0.7041 (4)	1.1530 (3)	0.0456 (8)	
H17A	0.3414	0.7742	1.1886	0.055*	
C16	0.6002 (5)	0.7336 (5)	1.3018 (3)	0.0617 (11)	
H16A	0.5433	0.8023	1.3272	0.093*	
H16B	0.7090	0.7806	1.2907	0.093*	
H16C	0.6073	0.6622	1.3557	0.093*	
C1	0.2101 (6)	1.0583 (5)	1.4017 (3)	0.0672 (11)	
H1A	0.3178	1.0496	1.4351	0.101*	
H1B	0.1304	1.0154	1.4472	0.101*	
H1C	0.2066	1.1573	1.3941	0.101*	
C18	0.5749 (5)	0.5542 (5)	1.1512 (4)	0.0654 (11)	
H18A	0.4883	0.4937	1.1028	0.098*	
H18B	0.6171	0.4988	1.2089	0.098*	
H18C	0.6616	0.5955	1.1104	0.098*	

### Atomic displacement parameters (Å^2^)

**Table:**

	*U*^11^	*U*^22^	*U*^33^	*U*^12^	*U*^13^	*U*^23^
Cu1	0.0362 (2)	0.0507 (3)	0.0374 (2)	0.01501 (18)	−0.00696 (16)	−0.00787 (17)
Br1	0.0733 (3)	0.1088 (4)	0.0734 (3)	0.0549 (3)	−0.0096 (2)	−0.0330 (3)
O2	0.0385 (13)	0.0546 (14)	0.0435 (12)	0.0180 (11)	−0.0066 (10)	−0.0112 (10)
O3	0.0419 (13)	0.0591 (15)	0.0408 (12)	0.0209 (11)	−0.0079 (10)	−0.0123 (10)
N1	0.0389 (15)	0.0411 (15)	0.0336 (13)	0.0104 (12)	−0.0023 (11)	−0.0008 (11)
C8	0.0374 (17)	0.046 (2)	0.0449 (18)	0.0123 (15)	−0.0077 (14)	−0.0012 (15)
N2	0.0387 (15)	0.0393 (15)	0.0375 (14)	0.0080 (12)	−0.0067 (11)	−0.0072 (11)
O4	0.0599 (16)	0.0672 (18)	0.0486 (15)	0.0250 (14)	−0.0163 (12)	−0.0091 (12)
O1	0.0450 (14)	0.0676 (17)	0.0441 (13)	0.0160 (12)	−0.0094 (11)	−0.0157 (12)
C7	0.0384 (17)	0.0368 (17)	0.0360 (16)	0.0086 (14)	0.0018 (13)	−0.0004 (13)
C5	0.0402 (19)	0.059 (2)	0.0440 (19)	0.0185 (16)	−0.0023 (15)	−0.0039 (16)
C2	0.0405 (18)	0.048 (2)	0.0374 (17)	0.0088 (15)	−0.0014 (14)	−0.0027 (14)
C15	0.043 (2)	0.061 (2)	0.047 (2)	0.0170 (17)	−0.0022 (15)	−0.0081 (16)
C9	0.0331 (16)	0.0435 (19)	0.0334 (16)	0.0037 (14)	−0.0015 (13)	0.0015 (13)
C10	0.0389 (18)	0.0436 (19)	0.0354 (16)	0.0040 (15)	0.0006 (13)	−0.0007 (14)
C3	0.050 (2)	0.049 (2)	0.0398 (18)	0.0120 (16)	0.0042 (15)	−0.0070 (15)
C13	0.071 (3)	0.083 (3)	0.045 (2)	0.020 (2)	−0.0027 (19)	−0.022 (2)
C11	0.046 (2)	0.046 (2)	0.0379 (17)	0.0096 (16)	−0.0007 (15)	0.0021 (14)
C4	0.048 (2)	0.054 (2)	0.051 (2)	0.0195 (17)	0.0041 (16)	−0.0053 (16)
C12	0.062 (3)	0.069 (3)	0.040 (2)	0.009 (2)	−0.0111 (17)	−0.0053 (18)
C6	0.0348 (17)	0.0389 (18)	0.0440 (18)	0.0088 (14)	0.0004 (13)	−0.0017 (14)
C14	0.063 (3)	0.076 (3)	0.056 (2)	0.024 (2)	0.0040 (19)	−0.019 (2)
O5	0.0379 (13)	0.0562 (15)	0.0488 (13)	0.0164 (11)	−0.0074 (10)	−0.0079 (11)
N3	0.0310 (14)	0.0506 (18)	0.0537 (17)	0.0044 (12)	−0.0062 (12)	−0.0006 (13)
C17	0.0346 (18)	0.049 (2)	0.050 (2)	0.0069 (15)	−0.0017 (15)	0.0024 (16)
C16	0.047 (2)	0.070 (3)	0.060 (2)	0.0083 (19)	−0.0155 (18)	−0.0032 (19)
C1	0.061 (3)	0.077 (3)	0.055 (2)	0.010 (2)	−0.014 (2)	−0.022 (2)
C18	0.045 (2)	0.076 (3)	0.077 (3)	0.026 (2)	−0.003 (2)	−0.005 (2)

### Geometric parameters (Å, º)

**Table:**

Cu1—O2	1.874 (2)	C9—C10	1.470 (4)
Cu1—N1	1.907 (3)	C10—C11	1.399 (4)
Cu1—O3	1.936 (2)	C3—C4	1.398 (5)
Cu1—O5	1.948 (2)	C3—H3A	0.9300
Br1—C4	1.899 (4)	C13—C14	1.364 (6)
O2—C7	1.294 (4)	C13—C12	1.365 (6)
O3—C9	1.282 (4)	C13—H13A	0.9300
N1—C8	1.281 (4)	C11—C12	1.385 (5)
N1—N2	1.386 (3)	C12—H12A	0.9300
C8—C6	1.429 (4)	C14—H14A	0.9300
C8—H8A	0.9300	O5—C17	1.262 (4)
N2—C9	1.326 (4)	N3—C17	1.284 (4)
O4—C11	1.359 (4)	N3—C18	1.446 (5)
O4—H4A	0.8200	N3—C16	1.454 (5)
O1—C2	1.369 (4)	C17—H17A	0.9300
O1—C1	1.414 (4)	C16—H16A	0.9600
C7—C6	1.418 (4)	C16—H16B	0.9600
C7—C2	1.426 (4)	C16—H16C	0.9600
C5—C4	1.345 (5)	C1—H1A	0.9600
C5—C6	1.412 (5)	C1—H1B	0.9600
C5—H5A	0.9300	C1—H1C	0.9600
C2—C3	1.358 (5)	C18—H18A	0.9600
C15—C14	1.367 (5)	C18—H18B	0.9600
C15—C10	1.382 (5)	C18—H18C	0.9600
C15—H15A	0.9300		
			
O2—Cu1—N1	93.33 (10)	O4—C11—C12	117.5 (3)
O2—Cu1—O3	174.87 (9)	O4—C11—C10	122.6 (3)
N1—Cu1—O3	81.62 (10)	C12—C11—C10	119.8 (3)
O2—Cu1—O5	91.67 (10)	C5—C4—C3	122.0 (3)
N1—Cu1—O5	172.69 (11)	C5—C4—Br1	119.4 (3)
O3—Cu1—O5	93.28 (9)	C3—C4—Br1	118.7 (3)
C7—O2—Cu1	127.5 (2)	C13—C12—C11	120.0 (4)
C9—O3—Cu1	110.17 (19)	C13—C12—H12A	120.0
C8—N1—N2	118.0 (3)	C11—C12—H12A	120.0
C8—N1—Cu1	127.4 (2)	C5—C6—C7	119.7 (3)
N2—N1—Cu1	114.56 (19)	C5—C6—C8	117.7 (3)
N1—C8—C6	124.1 (3)	C7—C6—C8	122.6 (3)
N1—C8—H8A	118.0	C13—C14—C15	119.5 (4)
C6—C8—H8A	118.0	C13—C14—H14A	120.2
C9—N2—N1	109.4 (2)	C15—C14—H14A	120.2
C11—O4—H4A	109.5	C17—O5—Cu1	122.1 (2)
C2—O1—C1	118.4 (3)	C17—N3—C18	121.9 (3)
O2—C7—C6	124.4 (3)	C17—N3—C16	121.1 (3)
O2—C7—C2	118.6 (3)	C18—N3—C16	117.0 (3)
C6—C7—C2	117.0 (3)	O5—C17—N3	123.4 (3)
C4—C5—C6	120.2 (3)	O5—C17—H17A	118.3
C4—C5—H5A	119.9	N3—C17—H17A	118.3
C6—C5—H5A	119.9	N3—C16—H16A	109.5
C3—C2—O1	124.7 (3)	N3—C16—H16B	109.5
C3—C2—C7	122.2 (3)	H16A—C16—H16B	109.5
O1—C2—C7	113.1 (3)	N3—C16—H16C	109.5
C14—C15—C10	121.6 (3)	H16A—C16—H16C	109.5
C14—C15—H15A	119.2	H16B—C16—H16C	109.5
C10—C15—H15A	119.2	O1—C1—H1A	109.5
O3—C9—N2	123.8 (3)	O1—C1—H1B	109.5
O3—C9—C10	119.6 (3)	H1A—C1—H1B	109.5
N2—C9—C10	116.5 (3)	O1—C1—H1C	109.5
C15—C10—C11	118.0 (3)	H1A—C1—H1C	109.5
C15—C10—C9	119.2 (3)	H1B—C1—H1C	109.5
C11—C10—C9	122.8 (3)	N3—C18—H18A	109.5
C2—C3—C4	118.9 (3)	N3—C18—H18B	109.5
C2—C3—H3A	120.6	H18A—C18—H18B	109.5
C4—C3—H3A	120.6	N3—C18—H18C	109.5
C14—C13—C12	120.9 (4)	H18A—C18—H18C	109.5
C14—C13—H13A	119.6	H18B—C18—H18C	109.5
C12—C13—H13A	119.6		

### Hydrogen-bond geometry (Å, º)

**Table:**

*D*—H···*A*	*D*—H	H···*A*	*D*···*A*	*D*—H···*A*
O4—H4*A*···N2	0.82	1.84	2.566 (3)	146
